# Effect of a tailored sepsis treatment protocol on patient outcomes in the Tikur Anbessa Specialized Hospital, Ethiopia: results of an interrupted time series analysis

**DOI:** 10.1186/s13012-022-01221-8

**Published:** 2022-07-19

**Authors:** Lisa M. Puchalski Ritchie, Lemlem Beza, Finot Debebe, Andualem Wubetie, Kathleen Gamble, Gerald Lebovic, Sharon E. Straus, Tigist Zewdu, Aklilu Azazh, Cheryl Hunchak, Megan Landes, Dawit Kebebe Huluka

**Affiliations:** 1 Department of Medicine, University of Toronto, Toronto, Canada; 2 Li Ka Shing Knowledge Institute, St. Michael’s Hospital, Toronto, Ontario M5B 1W8 Canada; 3 Department of Emergency Medicine, University Health Network, Toronto, Canada; 4 Institute of Health Policy, Management and Evaluation, University of Toronto, Toronto, Canada; 5 Department of Emergency Medicine, College of Health Sciences, Addis Ababa University, Addis Ababa, Ethiopia; 6 Division of Emergency Medicine, Department of Family and Community Medicine, University of Toronto, Toronto, Canada; 7 Department of Internal Medicine, College of Health Sciences, Addis Ababa University, Addis Ababa, Ethiopia

**Keywords:** Sepsis, Interrupted time series, Treatment protocol, Ethiopia, Africa

## Abstract

**Background:**

Despite improvement, sepsis mortality rates remain high, with an estimated 11 million sepsis-related deaths globally in 2017 (Rudd et. al, Lancet 395:200-211, 2020). Low- and middle-income countries (LMICs) are estimated to account for 85% of global sepsis mortality; however, evidence for improved sepsis mortality in LMICs is lacking. We aimed to improve sepsis care and outcomes through development and evaluation of a sepsis treatment protocol tailored to the Tikur Anbessa Specialized Hospital Emergency Department, Ethiopia, context.

**Methods:**

We employed a mixed methods design, including an interrupted times series study, pre-post knowledge testing, and process evaluation. The primary outcome was the proportion of patients receiving appropriate sepsis care (blood culture collection before antibiotics and initiation of appropriate antibiotics within 1 h of assessment). Secondary outcomes included time to antibiotic administration, 72-h sepsis mortality, and 90-day all-cause mortality. Due to poor documentation, we were unable to assess our primary outcome and time to antibiotic administration. We used segmented regression with outcomes as binomial proportions to assess the impact of the intervention on mortality. Pre-post knowledge test scores were analyzed using the Student’s *t*-test to compare group means for percentage of scenarios with correct diagnosis.

**Results:**

A total of 113 and 300 patients were enrolled in the pre-implementation and post-implementation phases respectively. While age and gender were similar across the phases, a higher proportion (31 vs. 57%) of patients had malignancies in the post-implementation phase. We found a significant change in trend between the phases, with a trend for increasing odds of survival in the pre-implementation phase (*OR* 1.24, 95% CI 0.98–1.56), and a shift down, with odds of survival virtually flat (*OR* 0.95, 95% *CI*. 0.88–1.03) in the post-implementation phases for 72-h mortality, and trends for survival pre- and post-implementation are virtually flat for 90-day mortality. We found no significant difference in pre-post knowledge test scores, with interpretation limited by response rate. Implementation quality was negatively impacted by resource challenges.

**Conclusion:**

We found no improvement in sepsis outcomes, with a trend for increasing odds of survival lost post-implementation and no significant change in knowledge pre- and post-implementation. Variable availability of resources was the principal barrier to implementation.

**Trial registration:**

Open Science Framework osf.io/ju4ga. Registered June 28, 2017

**Supplementary Information:**

The online version contains supplementary material available at 10.1186/s13012-022-01221-8.

Contributions to the literature

- This is one of few studies to implement and evaluate an intervention and implementation strategy to improve clinical care and outcomes among sepsis patients tailored to the resource challenges of a LMIC healthcare setting.
- Variability in resource availability was identified as the primary barrier to implementation quality, sustainability, and scalability.
- Our findings highlight the need for system interventions; as that while tailoring to context can offer solutions to some barriers, it is unlikely to be sufficient to adequately address the resource constraints facing low-resource healthcare settings.

## Background

An estimated 49.8 million sepsis cases and 11 million sepsis-related deaths occurred globally in 2017 [1]. Despite improvement in recent decades [1] with increased focus on and development of evidence-based guidance for sepsis management, sepsis mortality rates remain high, with a recent meta-analysis of 51 studies predominantly from high-income countries (HICs) reporting hospital and intensive care unit (ICU)-treated sepsis mortality rates of 26.7% and 41.9%, respectively [2]. While data are limited, sepsis incidence and mortality rates are thought to be higher in low- and middle-income countries (LMICs), with an estimated 85% of global sepsis cases and sepsis mortality occurring in LMICs in 2017 [1], case fatality rates as high as 80% [3], and evidence of a similar trend for improved sepsis mortality in LMICs lacking [1].

While early recognition and treatment are known to improve sepsis outcomes [4], only 10–30% of patients globally receive optimal care such as appropriate antibiotics and resuscitation [5]. This gap is due in part to the intense resource demands of early sepsis treatment guidelines, found challenging even in HICs [6]. However, evidence from 3 recent randomized controlled trials found that the higher resource guideline components (such as central venous catheters) do not improve sepsis outcomes when compared with lower resource interventions, namely early recognition, and appropriate antibiotic and supportive care [1, 7–9]. These findings are promising and are consistent with the WHO 2011 recommendations for management of sepsis in LMICs [10].

While it is recognized that these key components of sepsis care are translatable to low-resource healthcare settings [11], studies where implementation of sepsis protocols in low-resource settings was found to increase mortality [12–14] highlight the need for development and evaluation of sepsis treatment algorithms tailored to context. Tailoring to context should ideally be based on local data and include considerations of differences in patient populations, causal pathogens, and antibiotic resistance patterns, as well as the human and material resource challenges of the setting in which the protocol is to be applied [15].

We aimed to address an identified gap in sepsis care, through development and evaluation of a sepsis treatment protocol tailored to the Tikur Anbessa Specialized Hospital Emergency Department (TASH-ED) context and through this to improve clinical care and outcomes among sepsis patients treated in the TASH-ED. In addition, we hoped to generate principles to inform efforts to address gaps in other areas of care in the TASH-ED, other LMICs, and in low-resource healthcare settings within HICs.

## Methods

### Design

Our overall project design employed a concurrent nested mixed methods design [16]. An interrupted times series (ITS) study, and pre-post knowledge testing, was used to evaluate the effectiveness of the tailored sepsis treatment protocol in improving care and reducing sepsis mortality. An interrupted time series design was chosen to provide a rigorous evaluation of the intervention effect, as randomization was not possible due to the risk of contamination with implementation at a single site. In addition, we completed a process evaluation (to be reported separately) using interviews and a document review, to assess implementation, to identify barriers to and facilitators of scalability and sustainability, and to assess the potential of the approach to address other common high burden clinical presentations in the TASH-ED and other LMICs and in low-resource health-care settings within HICs.

This study is part of a larger project informed by the knowledge-to-action framework [17], with prior work conducted to identify the gap [18] and to understand barriers and facilitators to knowledge use [19]. An integrated KT approach has been employed throughout this project with stakeholders and knowledge users, including frontline clinicians and trainees, department managers, and leadership, engaged in all aspects of the project [20]. Stakeholder and knowledge user engagement has included participation of stakeholders as co-investigators in formative studies [17, 18], in person meetings with leadership and frontline clinicians throughout project development, usability testing, and implementation. They were full project partners who brought context-specific experience and expertise, and their input was incorporated in each stage of the research.

### Setting and participants

The study was conducted in the TASH-ED. TASH is the largest publicly funded academic referral hospital and is owned by the Addis Ababa University (AAU). It is the site of the first emergency medicine (EM) residency program in Ethiopia and the Masters Nursing Program in Critical Care and EM. The TASH-ED has an estimated 20,000 patient visits annually, with 20–25% of patients’ critically ill and requiring emergent care. Sepsis is the second leading cause of mortality, accounting for 19% of deaths within 72 h of presentation to the TASH-ED [18]. Children ≤ 12 years of age and obstetrical emergencies are managed in other EDs. The TASH-ED is staffed by EM and off-service residents supervised by 6 EM faculty. The majority of nursing staff have bachelor’s degree training.

All TASH-ED staff and trainees rotating through the department during the implementation and post-implementation period were introduced to the study and sepsis protocol and encouraged to participate. All patients with suspected or proven sepsis presenting for care during the study period were eligible for inclusion and approached to participate.

### Intervention

#### Sepsis protocol development

The surviving sepsis campaign [21] led the development of evidence-based guidance for sepsis management in LMICs [10, 19, 22, 23]. These guidelines formed the basis of our sepsis management protocol with adaption to the TASH-ED context, based on our preparatory data including a prospective cohort study of mortality patterns and qualitative study of barriers to and facilitators of development and utilization of evidence-based clinical algorithms in The TASH-ED [18, 24], stakeholder consultation, and recent TASH-ED data on pathogens and antimicrobial sensitivity [25].

**Fig. 1:**
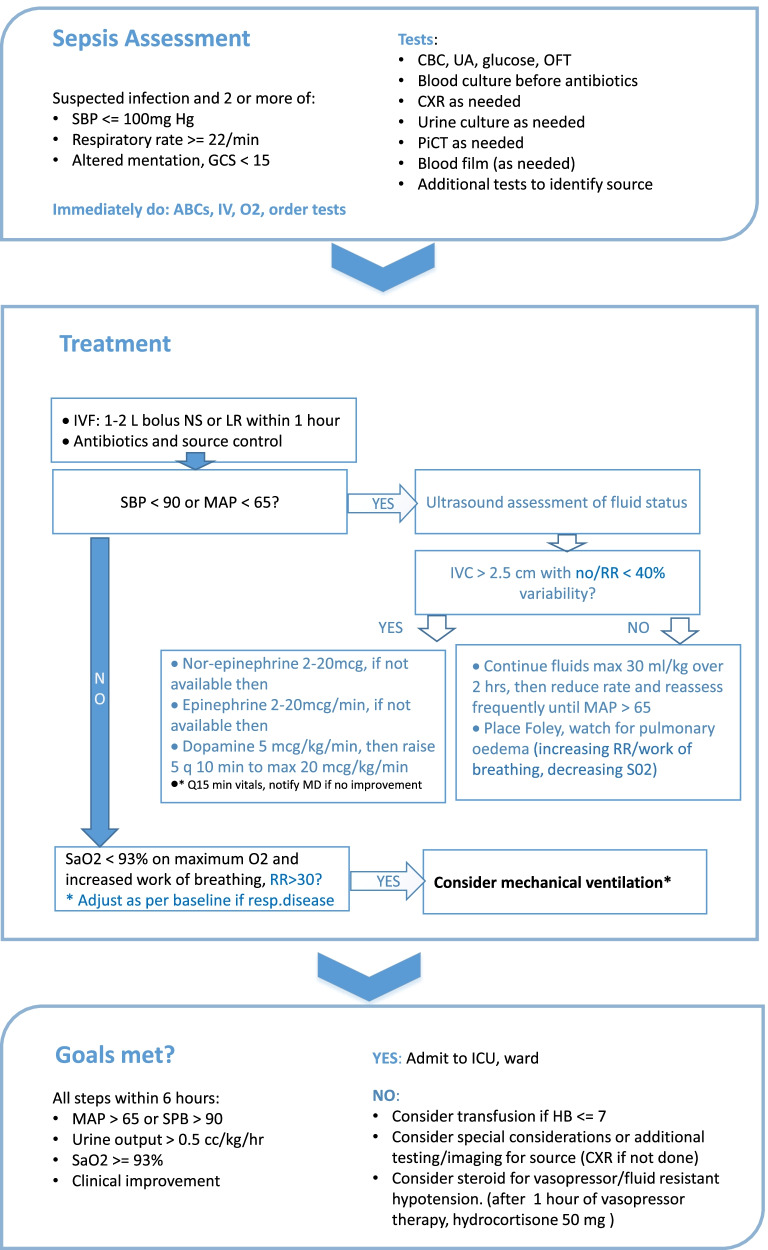
Sepsis protocol flow chart

The protocol includes a flow chart beginning with diagnostic criteria, followed by a stepwise approach to management of patients meeting diagnostic criteria (Fig. 1). Sepsis was diagnosed as suspected or confirmed infection and the presence of 2 or more quick sepsis-related organ failure assessment (qSOFA) criteria [26]. In order to facilitate early recognition and intervention, a trigger was posted at triage, with triage nursing staff asked to flag and notify a physician if the triage criteria were met. The protocol included standard components, such as obtaining blood cultures before initiation of antibiotics, fluid resuscitation, and early initiation of antibiotic therapy. Management was adapted to common pathogens and antibiotic sensitivities based on recent local data, included first- and second-line recommendations to address medication availability and patient ability to pay, and provided a reminder to consider and modify treatment for proven/or suspected tuberculosis and/or malaria. Additionally, as ICU capacity is limited at TASH, in order to avoid over-resuscitation necessitating ventilator support unnecessarily, ultrasound assessment of fluid status conducted by senior clinicians was incorporated into the protocol to guide fluid administration and initiation of vasopressor therapy.

The protocol was circulated widely to emergency medicine and ICU physicians and senior nurses based at or with experience working at TASH, and revised iteratively, with input from a human factors engineer with expertise in user-centered design throughout the process. The protocol was then usability tested with TASH-ED clinical staff with a range of clinical training and experience. Usability testing conducted in person employed a think out loud approach with participants asked to use the protocol to manage 3 clinical scenarios, with the PI (LPR) and a research assistant (RA) observing and taking notes. At the end of the think-aloud process, participants were asked to rate the tool using the System Usability Scale [27] and probed regarding any queries that arose during observations of the think-aloud process. Three rounds of usability testing were conducted with scores ranging from 80 to 87.5, with 3 consecutive scores above 68 considered above average [25].

#### Implementation strategy

**Fig. 2:**
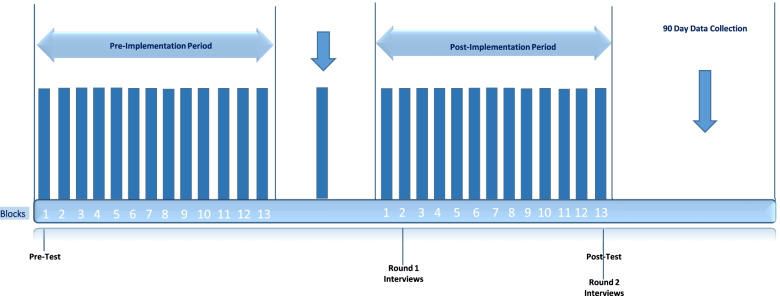
Study design and flow. Legend:  = 4 weeks

Study design and flow are outlined in Fig. 2. The pre-implementation phase began on June 26, 2017, and included 13 4-week blocks, during which no changes were made with care of sepsis patients left to the discretion of the provider. During the 4-week implementation phase (June 25 to July 22, 2018), the protocol (intervention) and implementation strategy were finalized and introduced to the staff. The post-implementation phase also consisted of 13 4-week blocks, from July 23, 2018, to July 20, 2019.

The implementation plan was developed by mapping identified barriers and facilitators to evidence-based implementation strategies. An initial list of barriers and facilitators to use of evidence-based protocols in the TASH-ED was developed based on barriers and facilitators identified in our previous study [24] and informed by the Theoretical Domains Framework [28] for individual level and Consolidate Framework for advancing implementation science (CFIR) [29] for context-level barriers/facilitators. The list was supplemented with sepsis-specific considerations through stakeholder consultation with TASH-ED staff from the range of clinical backgrounds and experience, as well as colleagues from Toronto with experience working in the TASH-ED. Barriers and facilitators identified through this process were mapped to implementation strategies using the COM-B and the CFIR-ERIC [30, 31], and the final strategy was selected based both on evidence for effectiveness and considerations of feasibility and sustainability (see Table 1).

**Table 1:** Implementation strategies mapped to barriers/facilitators

Barriers/facilitators	Implementation strategies
**Capability**	
*Understanding of the burden of sepsis in TASH-ED*	*Educational meetings, included local sepsis data*
*Lack of fit of sepsis protocols with the TASH-ED context*	*Protocol adapted to local antibiotic sensitivities and special considerations for TB/malaria and tailored to context*
*Protocol will act as a memory aid/reminder* *Hard to break “habits”*	*Hard copy reminders (posters and pocket card)*
**Opportunity**	
*Lack of human and material resources*	*Protocol includes alternative antibiotic choices to address medication availability, time to procurement, and patient ability to pay*
*Heavy workload due to high patient acuity and volumes*	*Protocol included a “triage trigger” to address high patient volumes, which could lead to delayed care*
*Lack of computer/Internet access*	*Hard copy reminders posted in acute care areas and pocket cards for easy reference*
*Modeling and endorsement by senior clinicians important for implementation success*	*Local opinion leader and senior clinicians, part of study/implementation team, and endorsed protocol and project during educational meetings*
**Motivation**	
*Belief that protocol is needed and will improve patient care and outcomes* *Belief that use of protocol will improve efficiency*	*Educational meetings: included local sepsis data, outlined evidence base of protocol including adaptation to local data, and tailoring to local resources*
*Concerns that resource barriers will limit implementation success* *Endorsement by leadership will support uptake*	*Local opinion leader and senior clinicians, part of study/implementation team, and endorsed protocol and project during educational meetings*

A detailed description of the sepsis protocol intervention and implementation strategy, which follows the template for intervention description and replication (TIDieR) format [32], appears in Table 2 (see TIDieR checklist, Additional file 1).

**Table 2:** Detailed description of sepsis protocol intervention and implementation strategy

	Sepsis protocol intervention
Rationale/goals	Intervention designed to address a recognized gap in sepsis care and through this improve patient outcomes. Specifically, the intervention was designed to support early identification of patients with suspected or confirmed sepsis and to provide a step-by-step guide to evidence-based clinical care of sepsis patients, tailored to the TASH-ED context
Materials and procedures	Intervention components included the following: knowledge adapted to local context, implementation strategies tailored to barriers to and facilitators of implementation, educational meetings, hard copy reminder tools, and local opinion leaders The protocol was developed based on existing evidence-based guidelines, with adaptation to the TASH-ED context based on local data regarding pathogens and antibiotic sensitivities and consideration of resource availability Implementation strategies were tailored to barriers to and facilitators of implementation, identified in our prior work and discussions with stakeholders regarding barriers/facilitators unique to or of specific importance to sepsis care in the TASH-ED. Examples of tailoring included the following: selection of hard copy reminders due to limited Internet access in the TASH-ED, inclusion of both ED leadership and a local opinion leader in educational and ad hoc meetings, and inclusion of alternative therapeutic options to address medication availability Educational meetings were conducted with TASH-ED clinical staff during the 4-week implementation phase. Sessions occurred during regularly scheduled physician and nursing meetings and began with a didactic session introducing the project, followed by an opportunity for questions and discussion. The didactic portion included presentation of goals and objectives of the project, TASH-ED sepsis mortality data and findings of the barriers/facilitator study that informed the implementation plan, description of the process for development of the sepsis protocol including tailoring to the local context, and step-by-step approach to care of patients with suspected or proven sepsis beginning with trigger for urgent assessment at triage. In addition, due to the high turnover of physician trainees rotating through the emergency department, a short power-point presentation was developed and presented at trainee orientation sessions. Hard copy reminder tools: three hard copy reminder tools were employed to support implementation. The first was a large poster, placed on the wall in the triage area. The poster included criteria to trigger a request by triage nursing staff for an urgent physician assessment for suspected sepsis. The second were large posters of the sepsis protocol, placed for easy reference in the resuscitation and acute care areas of the department. The third were laminated pocket cards, with the sepsis protocol on one side, and antibiotic recommendations and special considerations for tuberculosis and/or malaria, on the reverse side
Intervention provider	The TASH-ED is staffed by EM and off-service residents supervised by 6 EM faculty. The majority of nursing staff have bachelor’s degree training, with a small number of emergency medicine and critical care master’s degree nursing staff providing clinical care, coordinating activities in the ED, and teaching, coaching, and supervising students and junior nurses working the ED. Educational meetings were provided to both physician and nursing staff during the implementation period and through regular orientation sessions with new trainees rotating through the department. Sessions outlined the process for triggering an urgent physician assessment for suspected sepsis during time periods without a physician based on a triage. Sessions also highlighted the need for more frequent monitoring as part of the protocol sepsis, including the requirement for a senior resident to conduct the ultrasound assessment. EM residents receive training in and are highly skilled in ultrasound assessment, and the protocol used the volume assessment approach commonly employed in this setting, and therefore, no additional ultrasound training was provided
Method of delivery	Face to face
Location/context	TASH-ED is the largest publicly funded academic referral hospital in Addis Ababa and is the site of the first EM residency program in Ethiopia and the Masters Nursing Program in Critical Care and EM. A total of 20–25% of the estimated 20,000 patients treated annually in the TASH-ED are critically ill or injured, requiring emergent care. Given the small number of EM faculty, direct care is provided principally by EM and off-service residents, with supervision and support from EM faculty. A physician is based at triage weekdays during the day; at all other times, triage is staffed by 2 senior nurses. Lack of both human and material resources, and delays in accessing necessary resources, are common
Dose	Educational meetings were held at the start of the implementation period and during orientation sessions with new physician trainees rotating through the department on a biweekly/monthly basis. Posters remained posted throughout the implementation period. Pocket cards were distributed to new trainees rotating through the department during orientation
Tailoring	The protocol and implementation strategy were tailored to the TASH-ED context with no additional tailoring during implementation
Modifications	Initially, charts of suspected sepsis patients were to be flagged by placing a marker on the triage note and a physician informed of the need for an urgent assessment. However, as flagging was inconsistently done, nurses were asked to bring the triage note to the physician and inform them of the need for assessment. This adaptation was made during the first month post-implementation
Fidelity	Fidelity information was collected informally via study team meetings, intermittent attendance at nursing and physician meetings, site visits in the first and last quarter of the post-implementation period by the PI and Toronto-based research coordinator, and interviews in the first and last quarters of the post-implementation period Several challenges to intervention fidelity were encountered (1) Pocket card distribution and education of new trainees to the department were not consistently implemented, in part due to absences of individuals responsible for this task (2) Triage notification to physicians of suspected sepsis cases was infrequent (3) Several specific and uncommon resource challenges, such as shortage of oxygen delivery equipment, were encountered and limited strict adherence to the sepsis treatment protocol Several additional meetings were held with clinical staff during regularly scheduled meetings in an effort to address these challenges, as well as inadequate charting (noted throughout the post-implementation period) of time of assessment, blood culture, and first antibiotic. Meetings were led by the study PI during site visits, a local opinion leader, and/or the local study team leads and included both an educational component and time for discussion of the issues encountered and strategies for improvement

The implementation strategy initially included the following: adaptation of the evidence-based sepsis protocol to local pathogen and antibiotic sensitivities and resource availability, tailoring of the implementation strategies to context including engagement of local opinion leaders indentified through our earlier work to support implementation, and educational meetings and hard copy reminder tools. The protocol was introduced, and an education session was provided by the study PI at regularly scheduled in-person physician and nursing meetings during the 4-week implementation phase, with local leadership in attendance to encourage participation and address any questions or concerns arising. Sessions began with a didactic introduction to the project, followed by an opportunity for questions and discussion. The didactic portion included presentation of goals and objectives of the project, TASH-ED sepsis mortality data and findings of the barriers/facilitator study that informed the implementation plan, description of the process for development of the sepsis protocol including incorporation of local antibiotic resistance data and tailoring to the local context, and step-by-step approach to care of patients with suspected or proven sepsis beginning with trigger for urgent assessment at triage. Given the high turnover of physician trainees rotating through the emergency department, a short power-point presentation was developed and presented during trainee orientation sessions. Additional meetings were held in conjunction with weekly staff meetings late in the first and last quarters of the implementation phase during visits from the Toronto-based study team, and ad hoc as needed, tailored to assess and address implementation challenges as needed. Endorsement by local opinion leaders was noted during all meetings, and they attended when available.

Three hard copy clinical reminder tools were developed and employed to support implementation. The first was a large poster (approximately 4 by 3 ft), placed on the wall in the triage area. The poster included criteria to trigger notification of the physician team for urgent assessment for suspected sepsis. The second were large posters (approximately 3 by 5 ft) of the sepsis protocol, placed for easy reference in the resuscitation and acute care areas of the department. The third were laminated pocket cards, with the sepsis protocol on one side, and antibiotic recommendations and special considerations for tuberculosis and malaria, on the reverse side.

### Data collection and outcome measures

The primary ITS outcome was the proportion of patients receiving appropriate sepsis care, defined as blood culture collection before antibiotics and initiation of appropriate antibiotics within 1 h of clinical assessment. Secondary outcomes included the following: time to antibiotic administration, early sepsis mortality (within 72 h of presentation), and 90-day all-cause mortality.

ITS outcomes were abstracted from patient charts (triage, physician and nursing notes), by local nursing staff trained as RAs and supervised by the local study team, using a standardized data collection form to identify and follow sepsis cases to hospital discharge. 90-day mortality was assessed from patient charts for admitted patients and by telephone for those discharged before 90 days. Calls to patients or their alternate contact, provided at the time of enrolment, continued for up to 2 weeks beginning at 90-day post-presentation. Due to turnover of the data collection team during the study period, double data entry was not possible. As patient records are paper based, charts were digitized to allow for verification of abstracted outcome data by a second study team member, with all abstracted outcome data reviewed and verified by a second study team member.

Sepsis knowledge was assessed using a brief paper-based questionnaire at the start of the pre-implementation period and at the end of the post-implementation period. The knowledge test was developed collaboratively with EM faculty members of the study team, using common clinical presentations to develop clinical scenarios representing a range of sepsis and non-sepsis clinical presentation. The knowledge test was introduced during regular staff meetings and available for pick up at the meetings and in a folder next to a locked drop box on the wall in the ED near the education center and rest areas. Reminders were given weekly for 2 weeks at regular staff meetings. Basic demographic data including clinical role, training, and years experience was collected at the start of the knowledge test, followed by the standardized clinical scenarios used to assess recognition and management. Participants were asked to make a unique mark on their paper to allow linking of pre- and posttests for analysis. Consent for participation in the pre-post knowledge test was implied by completion of the test.

Process evaluation data (unpublished data) was collected throughout the study using qualitative methods and included interviews with TASH-ED staff and a document review of study team notes. The detailed methods and findings of the process evaluation will be reported separately, with only key challenges to implementation and collection of ITS outcomes reported here to provide context to the ITS findings.

### Consent/ethics approval

Written consent was obtained from patients/guardians (for patients < 18 years of age) or substitute decision-makers (for patients deemed incompetent by the treating physician) and assent obtained from patients < 18 years of age at the time of enrolment. For participants initially incapable, consent was obtained directly from patients in person if they became competent prior to discharge or by telephone at 90-day follow-up as appropriate.

### Sample size estimation

Sample size for interrupted time series analysis is derived by the number of time points. At study initiation, we estimated that < 10% of sepsis patients received optimal care. We aimed to improve this by at least 10% (double the baseline rate selected by knowledge users as a clinically meaningful level of improvement). The precision of our estimates and the power of ITS in detecting the protocols impact depend on how precise the primary outcome is estimated and indirectly on the number of sepsis patients within the 2-week block.

Based on a pre-study estimate of 10–15 sepsis cases per week, the primary outcome was estimated with 12% margin of error for a 2-week block and 8% margin of error for a 4-week block. For an ITS analysis based on biweekly data, 44 time points provide > 95% power in detecting a mean difference of 10% in proportion of sepsis patients receiving optimal care between pre- and post-implementation phases. Analysis based on monthly data (22 months, 11 months in each phase) provides 90% power in detecting 10% increase in optimal care of sepsis patients.

We estimate that approximately 50% of the 100 TASH-ED staff regularly care for patients in a clinical capacity; with an estimated 50% pre-post testing participation rate, we expected 25 clinicians to complete the pre-post knowledge survey at each of the 2 time points.

### Analysis

#### Interrupted time series

Patients were categorized into pre-implementation, and post-implementation periods according to ED visit date. As the number of patients within the 2-week blocks was too limited to validly estimate the proportions, the primary outcome was summarized monthly, with 13 4-week blocks before and after implementation included in the analysis.

Descriptive statistics of patient characteristics (age, sex, comorbidities) were calculated pre- and post-implementation.

Due to poor charting of time to antibiotic, our primary outcome and time to antibiotic administration could not be reliably assessed or analyzed.

Autocorrelation was assessed using autocorrelation and partial autocorrelation plots and the Durbin-Watson test and found not to be significant for either secondary outcome. Segmented regression with outcome as a binomial proportion without inclusion of an autoregressive term was therefore used to assess the impact of the intervention on 72-h sepsis mortality and 90-day all-cause mortality. Inclusion of covariates was precluded by the low number of cases per time block. In addition, we conducted a worst-case sensitivity analysis with missing outcomes included as dead (felt to be the most likely outcome) to assess the potential impact of missing outcomes.

#### Pre-post knowledge test

Pre-post knowledge test scores were analyzed using the Student’s *t*-test to compare group means for percentage of scenarios with correct diagnosis.

#### Process evaluation

Interviews and study team notes were analyzed independently by at least two study team members, using qualitative content analysis [33], with preliminary analyses occurring concurrent with data collection.

## Results

### Interrupted time series results

**Fig. 3:**
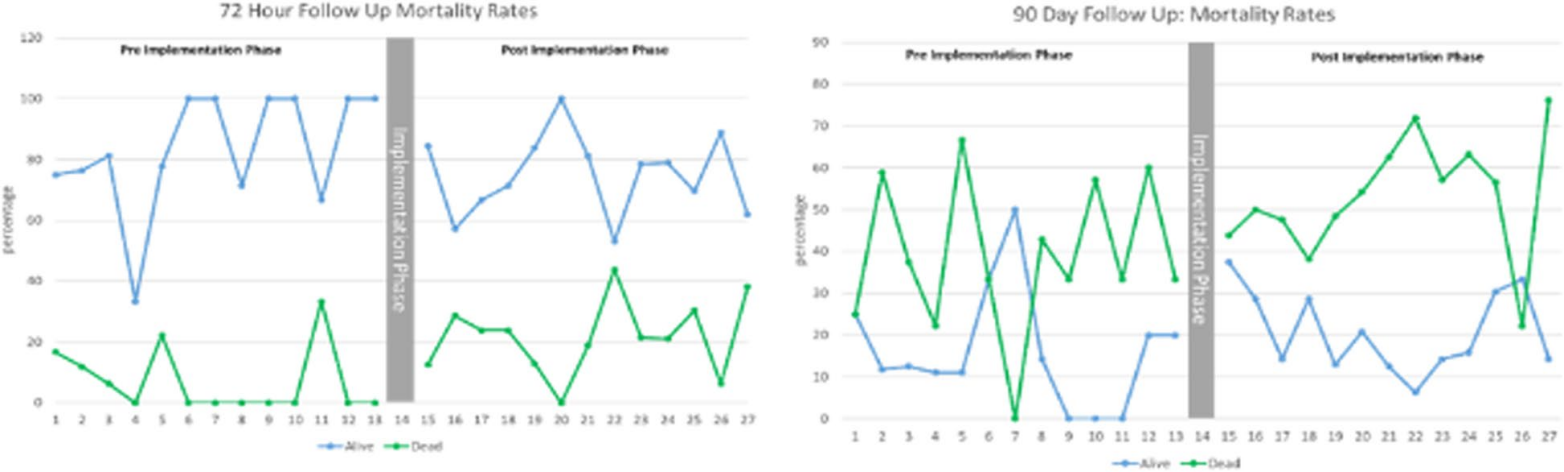
Patient outcomes by study time block and phase

A total of 113 and 300 patients were enrolled in the pre-implementation phase and post-implementation phase respectively. Only 3 patients were enrolled during the 4-week implementation phase and were therefore removed from the analysis. Patient characteristics by study phase are provided in Table 3. While age and gender were similar across the pre- and post-implementation phases, the proportions of patients with comorbidities are similar across the implementation phases, with the exception of malignancies which were higher in the post-implementation period (31 vs. 57%). Patient outcomes are provided in Table 4 and Fig. 3.

**Table 3:** Patient characteristics by study phase

	Pre-intervention 113	Post-intervention 300
**Age**	Age = 43.93, range 14–94	Age = 46.66, 12–95
**Gender**	M 41 (36%)	M 137 (46%)
	F 66 (58%)	F 163 (54%))
	Missing info 6 (5%)	
**TB**	4/113 (3.5%)	14/300 (4.6%)
**HIV**	8/113 (7.1%)	23/300 (7.7%)
**Malignancies**	35/113 (31%)	172/300 (57.3%)
**Heart disease**	10/113 (8.8%)	19/300 (8.3%)
**Kidney disease**	4/113 (3.5%)	3/300 (1%)
**Lung disease**	3/113 (2.7%)	13/300 (4.3%)

**Table 4:** Patient 72-h and 90-day outcomes by study phase

Study phase	72-h follow-up	90-day follow-up
Pre-implementation (113 participants)	Alive 92/113 (81.4%)	Alive 47/113 (41.6%)
	Dead 8/113 (7.1%)	Dead 48/113 (42.5%)
	Missing 13/113 (11.5%)	Missing 48/113 (42.5%)
Post-implementation (300 participants)	Alive 226/300 (75.3%)	Alive 61/300 (20.3%)
	Dead 65/300 (21.7%)	Dead 161/300 (53.7%)
	Missing 9/300 (3.0%)	Missing 78/300 (26.0%)

As noted above, due to poor charting with respect to time of blood culture and antibiotic administration (making determination of timing of blood culture relative to antibiotic administration unreliable), our primary outcome of appropriate sepsis care and secondary outcome of time to antibiotic administration could not be assessed.

### 72-h outcome

A total of 393 of the 413 (95%) patients enrolled in the study contributed 72-h outcome data, 102/113 (90%) in the pre-implementation phase and 291/300(97%) in the post-implementation phase. Findings of the segmented regression analysis with binomial proportions are summarized in Table 5. As the interaction term (interaction of slope and time block, labeled phase in the results tables) was significant, it was retained in the model. Analysis showed a significant change in trend between the time periods (*p* = 0.03), with a trend for a month over month increasing odds of patient survival in the pre-implementation phase (*OR* 1.24, 95% *CI* 0.98 1.56), and a shift down, with odds of survival in the post-implementation being virtually flat over time (*OR* 0.95, 95% *CI* 0.88 1.03).

**Table 5:** Results of segmented regression analysis of 72-h sepsis outcomes

	*OR*	Low. CI	Up. CI	*p*-value
Intercept	79.94	6.76	945.7	0.00
Phase	0.06	0.00	0.78	0.03
Time 1	1.24	0.98	1.56	0.07
Time 3	0.95	0.88	1.03	0.19

### 90-day outcome

A total of 290 of the 413 (70%) patients enrolled in the study contributed 72-h outcome data, 68/113 (60%) in the pre-implementation phase and 222/300 (74%) in the post-implementation phase. Findings of the segmented regression analysis with binomial proportions are summarized in Table 6. The interaction term (interaction of slope and time block, labeled phase in the results tables) was not significant and was therefore removed from the model. Analysis showed that the trend for survival pre- and post-implementation was virtually flat.

**Table 6:** Results of segmented regression analysis of 90-day outcomes

	*OR*	Low. CI	Up. CI	*p*-value
Intercept	0.37	0.13	1.07	0.07
Phase	1.47	0.43	5.05	0.54
Time 1	1.00	0.89	1.14	0.96
Time 3	0.94	0.87	1.02	0.16

### Sensitivity analysis

Sensitivity analysis with missing outcomes for patients included as deceased was conducted to assess the potential impact of missing outcome data on our findings. Results of the sensitivity analysis with missing outcomes included as deceased were essentially the same as the primary analysis (see Tables 7 & 8). Again, there is a significant change in trend pre- and post-implementation with a trend for increasing survival before implementation, and odds of survival were virtually flat after the intervention for 72-h survival, and no significant change in trend pre- and post-implementation with odds of survival is virtually flat in both periods for 90-day mortality.

**Table 7:** Sensitivity analysis results for 72-h outcomes

	*OR*	Low. CI	Up. CI	*p*-value
Intercept	52.97	7.94	353.23	0.00
Phase	0.06	0.01	0.47	0.01
Time 1	1.30	1.09	1.55	0.00
Time 3	0.98	0.91	1.05	0.58

**Table 8:** Sensitivity analysis results for 90-day outcomes

	*OR*	Low. CI	Up. CI	*p*-value
Intercept	0.22	0.08	0.60	0.00
Phase	1.47	0.46	4.70	0.52
Time 1	1.02	0.91	1.14	0.76
Time 3	0.96	0.89	1.04	0.32

### Pre-post knowledge test results

Average scores on recognition of sepsis pre- and post-implementation for physicians, nurses, and combined were as follows: 4.9 and 4.3 out of 7, 2.5 and 2.7 out of 7, and 4.1 and 3.0 out of 7, respectively. The lower overall score for the post-implementation period reflects the imbalance in physician and nurse participants, with physicians generally having higher scores, between the two periods. Analysis of pre-post knowledge tests found no significant difference between beginning and end of implementation for any group: physicians (*T* = 0.51, *p* = 0.62), nurses (*T* = −0.29, *p* = 0.76), and combined (*T* = 1.53, *p* = 0.14). As we did not identify any participants who completed the knowledge test in both time periods, change scores could not be calculated.

### Implementation challenges

Our concurrent process evaluation identified a number of barriers impacting implementation quality. Poor documentation, particularly with respect to time of clinical activities, including time of antibiotic administration was noted early in the study. Efforts to improve documentation through engagement via staff meetings with local leadership and opinion leaders present were made at several points during the study, with little change in documentation noted. The principal barrier to implementation was lack of resources essential to implementation of the protocol, including the following: lack of oxygen delivery equipment which was noted to start early in and to continue throughout the post-implementation phase, intermittent challenges with access to the ultrasound equipment due to a need for repairs, lack of and/or delays in accessing first-line medications, intermittent shortages of blood culture bottles, and lack of sufficient basic monitoring equipment (i.e., blood pressure cuffs) to allow for frequent monitoring, particularly when patient volumes were high. Additional barriers of note included the following: lack of uptake of the triage trigger process among nursing staff designed to reduce delays in physician assessment and treatment initiation, intermittent challenges in onboarding new trainees rotating through the department as a result of implementation team absences/staffing changes and poor documentation of time blood culture taken and first antibiotic administration making assessment of care processes, and audit and feedback to improve processes impossible.

## Discussion

We found that an evidence-based sepsis protocol tailored to the TASH-ED context did not improve sepsis mortality, with interpretation of our findings limited by a variety of factors outlined below. In fact, a trend for improved 72-h survival noted in the pre-implementation period was lost in the post-implementation period, and 90-day mortality outcomes remained flat across pre- and post-implementation periods. Sensitivity analysis to assess the impact of patients lost to follow-up yielded similar results. While it is possible that there is a true lack of effect, interpretation is limited by low-quality implementation primarily as a result of variable availability of resources essential for implementation and inconsistent on boarding of new trainees rotating through the department. In addition, poor documentation limited our ability to assess for factors that may be contributing to lack of impact. An additional potential contributing factor is an increased proportion of patients in the post-implementation phase with malignancies that are at increased risk of death in the setting of sepsis as a result of high rates of malnutrition and generally poor overall health status. The increased proportion of patients with malignancies is likely a result of redistribution of non-cancer patients to other centers as a result of expansion of trauma, toxicology, and general emergency care at other centers over this period, while the vast majority of cancer patients continued to be cared for at Tikur Anbessa. Finally, interpretation of pre-post knowledge testing is limited by a substantial imbalance in knowledge test participants between the two time periods and may represent an additional contributor to the lack of impact.

Despite the high burden of sepsis in LMICs, and recognized need for development and evaluation of evidence-based approaches to clinical care tailored to local context, relatively few studies addressing this gap in evidence have been published to date [3, 34, 35]. In addition, findings of studies to date have been somewhat mixed and have further highlighted the need for adaptation of evidence and clinical protocols to context.

Jacob et al.’s [22, 36] prospective before and after study of an early monitored sepsis management protocol conducted with adult sepsis patients in Uganda found significantly improved survival in the protocol management group relative to standard care. In addition, subgroup analysis found mortality in the protocol care group associated with baseline illness severity rather than volume of fluid received [9]. In contrast, Andrew’s et al.’s [12] pilot trial of modified early goal-directed therapy for adult sepsis patients in Zambia found no difference in inhospital mortality overall. Despite this, the study was stopped early due to a concern for an increased risk of mortality among patients in hypoxemic respiratory distress at baseline. A subsequent randomized trial of an early resuscitation conducted by Andrews et al. [13], with adult sepsis patients in Zambia, found increased inhospital mortality in the protocol arm relative to controls. Common among these studies were high rates of significant comorbidities, principally, high HIV prevalence of HIV (81–90%) with low CD4 (46–72) counts, and relatively high rates of TB infection (12–38%) [9, 12, 13].

In contrast to these studies, our study population had relatively low rates of HIV (8%) and TB (4.4%). While this might have been expected to improve odds of survival, our long-term mortality rates (42.5–53.7%) were similar to those of prior studies (33.0–64.2%). Due to relatively low numbers, we were unable to examine the impact of comorbidities on patient outcomes; however, it is likely that that relatively high proportion of patients with malignancies in particular (31% pre-implementation, 57% post-implementation) may have contributed to this finding.

A fourth study, by Maitland et al. [14], evaluated the impact of bolus fluids (albumin or saline) for children with severe febrile illness and impaired perfusion, in Kenya, Tanzania, and Uganda, and found increased 48-h and 4-week mortality among patients in both fluid bolus arms relative to controls. Unique to the pediatric sepsis study was a high incidence of malaria (57%) [14], which was much lower in the adult studies were reported (2–15%) [9, 12]. Of note, malaria cases had lower mortality rates compared to non-malaria cases, but did not differ in the impact of fluids on morality. Although authors note that the impact of aggressive fluid resuscitation was unclear, combined with the mixed results in the above noted adult sepsis studies, these findings raised concerns regarding aggressive fluid resuscitation in LMICs where ventilator support is limited or not available. Together, these findings have highlighted the need to tailor sepsis care protocols to the context and in particular to population characteristics, causal pathogens, and local resources, in order to optimize care and outcomes.

Studies of sepsis protocols in LMICs to date have largely used bedside assessment of volume status to direct care, including the following: symptoms or signs of respiratory distress [12], > = 3% decrease in oxygen saturation [9, 13], increase respiratory rate of > = 5 breaths per minute [9, 13], jugular venous pressure > = 3 cm above the sternal angle [9], or new crackles [13]. Dubin at al.’s [37] prospective cohort conducted in Argentina specifically evaluated the impact of dynamic tests of fluid responsiveness which included both noninvasive and invasive measures and found use of dynamic fluid assessment independently associated with reduced mortality.

Although available, capacity to provide ventilatory support is limited in the TASH-ED. Therefore, in order to address the potential risk of fluid overload with aggressive fluid resuscitation, we included early and regular clinical and ultrasound examinations of fluid volume to direct clinical care. Although approaches to and evidence for optimal use of ultrasound to assess volume are mixed [38], as invasive monitoring is not feasible in this setting, and ultrasound is routinely available and ED physicians well trained in its use, we included ultrasound fluid assessment by a senior clinician in our protocol. As noted, due to poor documentation, we were unable to assess changes in volume of fluid administration and/or changes in proportion of patients experiencing symptoms or signs of fluid overload pre- and post-implementation. Although discussions with TASH-ED supervising physicians and ICU staff noted no increase in sepsis patients requiring ventilator support post-implementation, this is a possible contributing factor given the increased proportion of cancer patients who are frequently malnourished [39] and at increased risk of pulmonary edema [13, 14]. Given the high rates of TB and Malaria in the above noted studies, and recognized importance of nonbacterial causes of sepsis and antibiotic resistance in LMICs [3, 15], our protocol included specific recommendations for TB and malaria and provided treatment recommendations based on local antibiotic resistance patterns. It is unclear if, how, and to what degree disease-specific recommendations were included in the sepsis protocols of the studies outlined above. As TB rates in our study population were low and no cases of malaria encountered, analysis of the use and impact of these specific recommendations was not possible.

Also unique to our study was the use of the qSOFA [26] to identify potential sepsis cases. At the time of development of our protocol, the qSOFA was relatively new and was therefore selected primarily based on its feasibility in the TASH-ED setting, where lactate (recommended by many protocols as part of septic workup) is expensive and not routinely available and turnaround of laboratory results could lead to important delays in initiating care. Several studies conducted in LMICs have since found the qSOFA to have good predictive ability [35, 40] and to reduce time to diagnosis [40]. Informal assessment of the application of qSOFA and accuracy of diagnosis in the first few months of implementation found the qSOFA was being appropriately applied, and that sepsis cases were being accurately captured.

Despite consideration of resources in tailoring our sepsis protocol to the TASH-ED context, including inclusion of alternative, second and in some cases third line, antibiotics and vasopressors, lack of essential resources presented an important barrier to implementation. Lack of resources is a widely recognized barrier to sepsis care in LMICs [3, 15] with a study by Abdu et al. [41] finding variable but substantial shortages in basic monitoring equipment, basic infrastructure, and antibiotics in both low- and middle-income countries. While tailoring to context can take account of and offer some solutions to addressing resource constraints, these efforts are likely to be insufficient alone, to address the high mortality burden of sepsis in LMICs.

### Strengths and limitations

This is one of relatively few studies to evaluate implementation of an evidence-based protocol for sepsis care tailored to local context in a LMIC setting. The strengths of this study include the following: tailoring of the protocol and implementation plan to the local context based on formative work to understand barriers and facilitators to implementation, recent local antibiotic resistance data, and the addition of ultrasound assessment of volume status to prevent fluid overload found to negatively impact outcomes in some studies of protocol-based sepsis care in LMICs [12–14]. In addition, our concurrent process evaluation (reported in detail separately) revealed important challenges and opportunities to improve implementation of the sepsis protocol and to inform development and implementation of evidence-based protocols for other high burden conditions in the TASH-ED.

The main study limitation is missing 90-day outcome data, with data available for only 60% and 74% of the pre- and post-implementation groups respectively. Additionally, limitations related primarily to implementation quality, which was negatively impacted by a variety of factors, including lack of essential resources, challenges with buy-in and documentation which limited our ability to monitor and address some aspects of implementation through audit and feedback, and staff and trainee turnover in general and implementation team in particular. While expanding the implementation team, and continued efforts to improve documentation, are likely to improve both implementation of the protocol and assessment of its impact, ongoing and emerging resource challenges will require sustained investment in essential resources.

## Conclusions

We found that an evidence-based sepsis protocol tailored to the TASH-ED context did not improve sepsis mortality, with a trend for improved 72-h survival lost in the post-implementation period and 90-day mortality outcomes remaining flat across pre- and post-implementation periods. In addition, we found no significant change in knowledge pre- and post-implementation. Interpretation is limited by a variety of factors including the following: low-quality implementation primarily as a result of lack of resources essential for implementation, poor documentation, and a relative low and imbalanced response rate to pre/post knowledge testing, limiting analysis. Further work to improve implementation and evaluate the impact of the sepsis protocol on clinical care and outcomes is needed. While tailoring of the intervention and implementation strategy to context can take account of and offer some solutions to some barriers. These efforts are, however, likely to be insufficient alone, to address the high mortality burden of sepsis in LMICs, if resource constraints are not adequately addressed.

## Supplementary Information

**Additional file 1.**TIDieR checklist.

## Data Availability

The datasets used and/or analyzed during the current study are available from the corresponding author on reasonable request.

## Abbreviations

EM: Emergency medicine
HICs: High-income countries
ICU: Intensive care unit
ITS: Interrupted time series
LMICs: Low- and middle-income countries
qSOFA: Quick sepsis-related organ failure assessment
PI: Principal investigator
RA: Research assistant
TASH-ED: Tikur Anbessa Specialized Hospital Emergency Department

## References

[1] Rudd KE, Johnson SC, Agesa KM, Shackelford KA, Tsoi D, Kievlan DR (2020). Global regional, and national sepsis incidence and mortality, 1990-2017: analysis for the Global Burden of Disease Study. Lancet.

[2] Marik PE (2014). Early management of severe sepsis: concepts and controversies. Chest.

[3] Schultz MJ, Dondorp AM, Dünser MW, Schultz MJ (2019). Current challenges in the management of sepsis in ICUs in resource-poor settings and suggestions for the future. Sepsis Management in Resource-limited Settings.

[4] Fleischmann-Struzek C (2020). Incidence and mortality of hospital- and ICU-treated sepsis: results from an updated and expanded systematic review and meta-analysis. Intensive Care Med.

[5] Kollef MH, Micek ST (2010). Using protocols to improve patient outcomes in the intensive care unit: focus on mechanical ventilation and sepsis. Sem Respir Crit Care Med.

[6] Daniels R (2011). Surviving the first hours in sepsis: getting the basics right. J Antimicrobial Chemother.

[7] Group, T.A.I.a.t.A.C.T (2014). Goal-directed resuscitation for patient with early septic shock. New Engl J Med.

[8] Mouncey PR (2015). Trial of early, goal-directed resuscitation for septic shock. N Engl J Med.

[9] Yealy DM (2014). A randomized trial of protocol-based care for early septic shock. N Engl J Med.

[10] Organization, W.H (2011). Integrated management of adolescent and adult illness: IMAI district clincian manual: hospital care for adolescents and adult guidelines for the management of common illnesses with limited resources.

[11] Becker JU, Theodosis C, Jacob ST, Wira CR, Grace NE. Surviving sepsis in low-income and middle-income countries: new directions for care and research. Lancet Infect Dis. 2009:577–82. 10.1016/S1473-3099(09)70135-5 19695494

[12] Andrews B (2014). Simplified severe sepsis protocol: a randomized controlled trial of modified early goal-directed therapy in Zambia. Crit Care Med.

[13] Andrews B (2017). Effect of an early resuscitation protocol on in-hospital mortality among adults with sepsis and hypotension: a randomized clinical trial. Jama.

[14] Maitland K (2011). Mortality after fluid bolus in African children with severe infection. N Engl J Med.

[15] Rudd KE (2018). The global burden of sepsis: barriers and potential solutions. Crit Care.

[16] Creswell JW, Plano Clark VL (2017). Designing and conducting mixed methods research.

[17] Graham ID (2006). Lost in knowledge translation: time for a map?. J Contin Educ Health Prof.

[18] Hunchak C (2015). Patterns and predictors of early mortality among emergency department patients in Addis Ababa, Ethiopia. BMC Res Notes.

[19] Kestler A (2013). The development of an emergency sepsis care algorithm in Botswana: Le développement un algorithme de prise en charge urgence des états septiques au Botswana. African J Emerg Med.

[20] Guide to knowledge translation planning at CIHR: integrated and end-of-grant approaches. 2022]; Available from: https://cihr-irsc.gc.ca/e/45321.html#a3.

[21] Surviving Sepsis Campaign. 2015; Available from: http://www.survivingsepsis.org/Pages/default.aspx.

[22] Jacob ST, Lim M, Banura P, Bhagwanjee S, Bion J, Cheng AC, et al. Integrating sepsis management recommendations into clinical care guidelines for district hospitals in resource-limited settings: the necessity to augment new guidelines with future research. BMC Med. 2013;107(11). 10.1186/1741-7015-11-107 PMC3635910 23597160

[23] Murthy S, Adhikari NK (2013). Global health care of the critically ill in low-resource settings. Ann Am Thorasic Soc.

[24] Puchalski Ritchie LM, Debebe F, Azazh A (2019). Barriers to and facilitators of the development and utilization of context appropriate evidence based clinical algorithms to optimize clinical care and patient outcomes in the Tikur Anbessa emergency department: a multi-component qualitative study. BMC Health Serv Res.

[25] Semret M, et al. Prolonged empirical antibiotic therapy is correlated with bloodstream infections and increased mortality in a tertiary care hospital in Ethiopia: bacteriology testing matters. JAC-Antimicrobial Resist. 2020;2(3). 10.1093/jacamr/dlaa039 PMC8210023 34240055

[26] Singer M (2016). The Third International Consensus Definitions for Sepsis and Septic Shock (Sepsis-3). JAMA.

[27] Gov., U., Improving the user experience: system usability scale (SUS). 2016.

[28] Michie S (2005). Making psychological theory useful for implementing evidence based practice: a consensus approach. Qual Saf Health Care.

[29] Damschroder LJ (2009). Fostering implementation of health services research findings into practice: a consolidated framework for advancing implementation science. Implement Sci.

[30] Michie S, van Stralen MM, West R (2011). The behaviour change wheel: a new method for characterising and designing behaviour change interventions. Implement Sci.

[31] Powell BJ (2015). A refined compilation of implementation strategies: results from the Expert Recommendations for Implementing Change (ERIC) project. Implement Sci.

[32] Hoffmann TC (2014). Better reporting of interventions: template for intervention description and replication (TIDieR) checklist and guide. Bmj.

[33] Hsieh H-F, Shannon SE (2005). Three approaches to qualitative content analysis. Qual Health Res.

[34] Bakhtawar S (2020). *Risk factors for postpartum sepsis: a nested case-control study*. BMC Pregnancy Childbirth.

[35] Rudd KE, Seymour CW, Aluisio AR (2018). Association of the quick sequential (sepsis-related) organ failure assessment (qSOFA) score with excess hospital mortality in adults with suspected infection in low- and middle-income countries. JAMA.

[36] Jacob ST, Banura P, Baeten JM, Moore CC, Meya D, Makiyingi L, et al. The impact of early monitored management on survival in hospitalized adult Ugandan patiens with severe sepsis: a prospective intervention study. Crit Care Med. 2012;40(7). 10.1097/CCM.0b013e31824e65d7 PMC3378757 22564958

[37] Dubin A (2020). Characteristics of resuscitation, and association between use of dynamic tests of fluid responsiveness and outcomes in septic patients: results of a multicenter prospective cohort study in Argentina. Ann Intens Care.

[38] Monnet X, Teboul JL (2018). Assessment of fluid responsiveness: recent advances. Curr Opin Crit Care.

[39] Gebremedhin TK (2021). Prevalence and risk factors of malnutrition among adult cancer patients receiving chemotherapy treatment in cancer center, Ethiopia: cross-sectional study. Heliyon.

[40] Shahsavarinia K, Moharramzadeh P, Arvanagi RJ, Mahmoodpoor A (2020). qSOFA score for prediction of sepsis outcome in emergency department. Pak J Med Sci.

[41] Abdu M, Wilson A, Mhango C, Taki F, Coomarasamy A, Lissauer D (2018). Resource availaility for the management of maternal sepsis in Malwai, other low-income countries, and lower-middle-income countries. Int J Gynecol Obstertr.

